# Protection of the Cervical Border

**Published:** 1885-01

**Authors:** Louis Ottofy

**Affiliations:** Ottofy, Dak.


### THE DENTAL REGISTER.

VOL. XXXIX.] JANUARY, 1885. [No. 1.

### COMMUNICATIONS.

### Protection of the Cervical Border.

BY LOUIS OTTOFY, D.D.S., OTTOFY, DAK.

The proper filling of the soft cervical border in difficult and inaccessible positions has probably given most of us more inconvenience than any other operation performed by the dentist. And especially do we find a great deal of trouble when using gold foil as a filling material in such places; oft times everything seems to work to entire satisfaction; the margin appears smooth, the gold seems thoroughly packed against the cervical wall, and everything indicates that the operation will be a success in every respect; and especially is this so after the finest excavator or probe, assisted with the strongest light, can not discover the slightest imperfection or flaw. But very often, to our dismay, in a short time such fillings fail at the very point where we exerted our best abilities, and hoped to utilize the advantages of our most perfect skill. There are several reasons why this is so; these reasons are divided into two classes: first, improper filling material; second, improper manipulation (supposed and apparent *carefulness*, to the contrary, notwithstanding).

The idea that in order to perform the grandest piece of artistic dentistry, we must have an unadulterated filling, as it were, an all gold filling—gold from beginning to end, is largely accountable for the first of these reasons. That discrimination in the selection of filling material is very essential is beautifully illustrated in the methods of filling root-canals, as formerly and now practiced. Some of our most prominent practitioners thought that the acme of dentistry was reached when they were able to manipulate gold with such skill that a tooth could be filled with it from the tip of its apex to the most exposed surface of its crown. A few years' practice readily determined that in order to fill a root canal properly, gold is probably one of the poorest materials at our command, and though it does not seem so high sounding, it proves more satisfactory when we can say, "this root is filled with gutta-percha." So is it, gentlemen, with the filling of cervical walls. Gold is one of the poorest substances, in many cases, to which we can resort as a filling material. Of course I am referring mainly to those frail, soft cervical borders extending somewhat under the gum. In most cases, where decay does not extend under the gum, where the enamel is hard, or at least medium, gold foil used alone proves very satisfactory, but it is almost always necessary when foil is so used, that it should be in the form of what is known as "soft foil;" the method of Dr. Black, of Jacksonville, Ill., is the best one I know of to obtain this foil in a perfectly "soft" condition. You can take any of the foils sold and known as "cohesive, semi-cohesive, or soft (non-cohesive,) "and keep it in a box containing, on a piece of cotton or spunk, a few drops of aqua-ammonia; this changes any of the above forms of foil into a very soft, fine, pliable and easily compressible foil; and if you take the trouble to examine the working of this gold with a magnifying glass while some one is using it, and then examine the cohesive foil under the same conditions, you will be astonished to see how differently they behave when packed against unyielding surfaces with an instrument. So, then, if you desire to use gold foil at the cervical margin, in fact adjoining any margin, use "soft" foil. In a great many instances other substances will prove to be much more satisfactory than gold, and one of these is tin foil, which, in very many cases, is the *ne plus ultra*, though sometimes it is quite difficult to manipulate; as regards union between it and gold, there is none other than a mechanical one, if serrated instruments are used; especially is tin foil preferable in such places where it is difficult to polish and finish the surfaces as gold must be finished; often a burnisher will give it sufficient smoothness, whereas gold, in my opinion, ought always to receive the usual finish of sandpaper and corundum paper disks at even the furthermost cervical border. Amalgam is another of the materials which proves very satisfactory in hidden, inaccessible cervical borders, especiallv in such, where for reasons well known to you, it is impossible to obtain a firm basis, wherefrom to build up with gold. In these cases I generally use Bonwill's quick-setting gold alloy, which is accompanied by a flask of re-distilled, or rather, by especially prepared mercury; sometimes I have found difficulty in obtaining sufficient anchorage in the amalgam for the gold, as this amalgam sets very quickly, and must be worked stiff; but I find less trouble by preparing the cavity in such a manner as to obtain sufficient anchorage for the gold, independent of the amalgam. Recently I have experimented some with what is known as "Sullivan's Cement," a package of which I received through the kindness of Dr. Harlan, of Chicago; he obtained it while in London; the preparation consists, I believe, of nothing but copper filings mixed with mercury, and which must be softened by heat, the surplus mercury pressed out with chamois skin. This amalgam, when hard, oxydizes very much and turns very black, and, consequently, must not be used in any but the second and third molars; the therapeutic effect of the oxyde is one of the points in its favor; my experience with it has, as yet, been limited, but sufficiently satisfactory to warrant its use.

The point involved in all these different manipulations is the use of a material which is adapted to the circumstances; as a general rule very soft, chalky teeth indicate the use of tin, and oftener amalgam, for the cervical border; medium and hard teeth, tin, and oftener "soft" gold.

As regards the second difficulty involved, improper manipulation, a few remarks will prove sufficient. In using any of these materials it is essential that they should be perfectly adapted to the borders of the cavity, which is difficult to do in some cases, with even these materials; fillings fail oftener at these points than anywhere else, for the least imperfection means final failure. After, and in fact, during the time of finishing fillings, they should be examined very carefully with a magnifying glass; the best of us will find remarkably rough edges and imperfections that the naked eye can not detect. I have often found it essential to spend some additional time on fillings which the naked eye had pronounced thoroughly finished.

